# Key Factors in Intervention Implementation, Fidelity, and Sustainability

**DOI:** 10.1093/geroni/igab046.2958

**Published:** 2021-12-17

**Authors:** Carey Sherman, Kate Gordon, Kenneth Hepburn

**Affiliations:** 1 Family Care Strategies, LLC, Ann Arbor, Michigan, United States; 2 Spilane Consulting, Columbia, Maryland, United States; 3 Emory University, Atlanta, Georgia, United States

## Abstract

As part of an NIA-supported effort to develop an online course to train individuals to lead the evidence-based Savvy Caregiver program and to orient sponsoring organizations to the program, we conducted semi-structured interviews to assess success and sustainability “best practices”. Interviews were conducted with 17 leaders and trainers from eleven Savvy-providing organizations. Analysis of these interviews identified two main themes associated with successful program implementation: leadership commitment and trainer ownership. Paramount to success appears to be leaders’ clear understanding of and enthusiastic commitment to the value of the Savvy program to the organization’s constituents. This translated to careful selection, training, management and on-going development of Savvy program trainers. It contributed to leaders’ appreciation of Savvy as a gateway for clients to seek out other programs and services from the organization, while the gathering of meaningful evaluation data (using established outcome-assessment instruments) contributed, in several cases, to garnering more lasting support to deliver the program. Organizations’ commitment to the program was demonstrated by securing the kinds of adequate and appropriate training, typically involving both instruction and modeling, for Savvy program leaders. These efforts fostered a sense of ownership among the leaders – the sense that the program had positive value for the caregivers served. These findings should be of interest both to scholars engaged in the development of interventions and for organizations implementing them. Taken together, the themes highlight several factors for program implementation that maximize the chances of maintaining fidelity to core program principles and ensuring its sustainability.

